# Green tea silver nanoparticles improve physiological motor and cognitive function in *BALB/c* mice during inflammation

**DOI:** 10.1016/j.heliyon.2023.e13922

**Published:** 2023-02-21

**Authors:** Herbert Izo Ninsiima, Ejike Daniel Eze, Kenneth Ssekatawa, Halima Nalugo, Caroline Asekenye, David Onanyang, Edson Ireeta Munanura, Moses Ariong, Kevin Matama, Gerald Zirintunda, Ngala Elvis Mbiydzenyuy, Fred Ssempijja, Adam Moyosore Afodun, Regan Mujinya, Ibe Michael Usman, Oscar Hilary Asiimwe, Julius Tibyangye, Keneth Iceland Kasozi

**Affiliations:** a Department of Physiology, School of Medicine, Kabale University, Box 317, Kabale, Uganda; b Department of Veterinary Pharmacy, Clinics and Comparative Medicine, School of Veterinary Medicine and Animal Resources, Makerere University, Box 7062, Kampala, Uganda; c Department of Anatomy, Faculty of Medicine, Mbarara University of Science and Technology, Box 1410 Mbarara, Uganda; d Department of Clinical Pharmacy and Pharmacy Practice, School of Pharmacy, Kampala International University Western Campus, Box 71, Bushenyi, Uganda; e Department of Biology, Faculty of Science, Gulu University, P.O. Box 166, Gulu, Uganda; f Department of Pharmacy, College of Health Sciences, Makerere University, Box 7062, Kampala, Uganda; g Department of Animal Production and Management, Faculty of Agriculture and Animal Sciences, Busitema University, Box 206, Soroti, Uganda; h Department of Basic Medical Sciences, Michael Chilufya Sata School of Medicine, Copperbelt University, Ndola, Zambia; i Faculty of Biomedical Sciences, Kampala International University Western Campus, Box 71, Bushenyi, Uganda; j Department of Anatomy and Cell Biology, Faculty of Health Sciences, Busitema University, Uganda; k School of Health Sciences, Mountains of the Moon University, Fort Portal, Uganda; l Marine Biodiscovery Centre, Department of Chemistry, University of Aberdeen, Old Aberdeen, AB243UE, Scotland, United Kingdom; m Department of Medical Laboratory Sciences, Faculty of Health Sciences, Muni University, P.O Box 725, Arua, Uganda; n Infection Medicine, Deanery of Biomedical Sciences, College of Medicine and Veterinary Medicine, University of Edinburgh, EH8 9JZ, Edinburgh, United Kingdom

**Keywords:** Small molecules, Uganda tea, The safety of tea, AgNPs, Physiology of nanoparticles, Green tea synthesis, silver nanoparticles, antibiotic resistance, Camellia sinensis, prunus africana

## Abstract

Information on the basic changes associated with green tea small molecules in acute inflammation is deficient. The purpose of the study was to characterize and establish the effects of green tea silver nanoparticles (AgNPs) following inflammation in *BALB/c* male mice. In this study, green tea silver nitrate nanoparticles were characterized and the extract were made up to constitute high (100%), medium (10%), and low (1%) concentrations for administration. Acute inflammation was induced in groups I–V of the experimental rodents by injecting 0.5 ml/kg of fresh egg albumin on the subplantar surface of the right hind paw and animals were monitored for 36 h. Group I–III were administered 100%, 10%, 1% green tea nanoparticles extract while group IV was given diclofenac. Group V was the positive control while group VI was the negative control that received the vehicle. Paw edema was measured at a 2 h interval for 3 days, while the pain was assessed by measuring the locomotion activity using the voluntary wheel running and the anxiety-like behavior. Hypersensitivity was measured through the temperature sensation experiment and a non-linear regression analysis was done. Here, synthesized green tea AgNPs registered an absorbance band at 460 nm, phytochemicals due to presence of organic functional groups of O=C=O of oxycarbons, of C=C of a conjugate alkene, C=O of a stretching bond of a secondary alcohol. The silver green tea nanoparticles were spherical, covered by a slimy layer, capped and stable. Green tea AgNPs significantly decreased temperature hypersensitivity in *BALB/c* male mice and this demonstrated their protective effects. Low concentrations of green tea nanoparticles inhibited edema thus mimicking effects of diclofenac, however, the percentage of inhibition was highest in medium and high silver-tea nanoparticles concentrations demonstration the importance of concentration in therapeutics. Anxiety was lowest in *BALB/c* male mice treated with high concentrations of silver green tea nanoparticles, and this led to increased locomotory activity in mice. Green tea AgNPs have strong anti-inflammatory effects at high concentrations. Concentrations of green tea AgNPs modulated basic sensory and motor behaviors in *BALB/c* male mice demonstrating their importance in complementary and integrative medical practice.

## 1 Introduction

Inflammation is a critical protective mechanism in the healing process and occurs in response to tissue and organ exposure to harmful stimuli such as mechanical, microbial pathogens, irritants, or toxic cellular components, response to elevated temperature (>38.0 °C) or hypothermia (<36.0 °C), [1]. Systemic changes in inflammation include tachycardia, tachypnea, leukocytosis (>12 × 109/L), or leucopenia [2]. In mice, inflammation has also been shown to involve immunological and molecular changes [3]. In acute inflammation, proinflammatory cytokines are responsible for tissue injury and damage, while levels of C-reactive protein (produced in the liver) are always elevated [4]. Resolution after inflammation involves leukocyte death and clearance of dead cells through pro-apoptotic pathways by increased expression of the ‘don’t eat me proteins' [5]. In the brain, elevated glucocorticoids which are pro-inflammatory, disrupt brain functioning in the hippocampus [3].

Inflammation involves pain, swelling (edema), temperature (heat), redness (increased blood supply), and loss of function. Pain is an unpleasant sensation resulting from real or potential damaged tissue and is one of the major signs of inflammation. This is caused by the accumulation of cytokines which are small secreted proteins released by cells under stress or when injured [6]. Certain cytokines or chemokines were proven to be involved in the initiation and the persistence of pathologic pain by directly interacting with nociceptive sensory neurons. Moreover, these inflammatory cytokines caused nerve-injury or inflammation-induced central sensitization and resulted in contralateral hyperalgesia [7].

Pain is associated with selling during inflammation [8], however, studies on the role of green tea nanoparticles in acute inflammation remain scarce. The degree of swelling is associated with pain due to pro-inflammatory cytokines i.e., interleukin (IL) IL-1, IL-6, and tissue necrosis factor-alpha (TNF-α) which lead to intravascular coagulopathies associated with an increase in size at the inflammatory foci [9]. Nanoparticles have been shown to protect have antioxidant and anti-inflammatory properties [10]. Tea particles have also been shown to have antioxidant, immunoregulatory, and cardiopulmonary protective effects [11]. Green nanoparticle synthesis has arisen from the continued demand for natural products use for complementary medicine [12]. Polyphenol nanoparticles are generated from green tea extraction after noncovalent self-assembly and covalent copolymerization [12]. These polyphenols are responsible for the reduction in swelling by regulating Ca^2+^ levels [13].

Swelling and pain often occur together and are associated with a decrease in exercise tolerance and function of the limb (palm) [14]. Locomotion involves the movement of mice from one place to another, however, the presence of pain sensation disrupts this normal physiological behavior i.e. on intensity and duration [15]. This subsequently raises the level of anxiety in the animal and causes nervousness, restlessness, fear of moving from one place to another, and pervades a sense of unrealistic worry about everyday life situations [15]. A mouse with an inflamed paw receiving an anti-inflammatory agent may move long distances as compared to one that has received a placebo [16]. Green tea polyphenols could mitigate oxidative stress [17], however studies limited studies have been conducted on acute pain and swelling.

Chronic inflammation and acute inflammation are the major classes of inflammation and chronic inflammation is associated with various degenerative diseases such as arthritis and atherosclerosis, allergies and malignancies [18]. Natural product-based drugs are preferred as a new therapeutic option for the prevention and treatment of inflammatory diseases [19,20]. Flavonoids are one of the various types of phytochemicals found in natural products; mainly vegetables and herbal medicines (see Ref. [11] for a comprehensive review and morin [21]). Using plant extracts like aqueous green tea extract is cheaper, more readily available, and has fewer side effects as compared to the conventional anti-inflammatory therapies and it can also improve an individual’s health [22].

Green tea has therapeutical effects against wounds as it promotes tissue remodeling and bone healing [23,24] as well as antibacterial activity (see Ref. [25] on cytotoxic antidiabetic effects i.e., protein kinase inhibition, antioxidant activity and α-amylase inhibition assays). Invitro studies have shown that green tea nanoparticles improve on keratin material and adhesiveness thus enhancing healing [24,26]. They also lessen the effective dose which is required to achieve therapeutical proficiency (see Ref. [27] adoption in cancer therapy) thus reducing the circulation of resistant genes in a population [28]. This is possible through the nano-potentiated absorption of drugs [29], making them the current major leading drug delivery options in therapeutics [30,31]. In this study, we investigated the basic Musculo-sensory pathway in male mice under green tea nanoparticle therapy.

## 2 Methods

### 2.1 Preparation of green tea extract-silver nitrate nanoparticles

Igara Tea farms in the Bushenyi district of southwest Uganda provided fresh green tea leaves (Camellia sinensis). They were scrubbed clear of dirt and lichens before being placed on drying tables with a fresh piece of paper in a shady area to dry. After being chopped or sliced into smaller pieces measuring 1–2 cm^2^, the plant materials were then ground into a powder using an electric laboratory grinder. Two separate extraction bottles containing 400 g of each powdered plant material each were filled with 1.5 L of deionized water for the extraction process. The mixtures were then allowed to steep for five days with occasional stirring twice a day to ensure homogenous mixing and extraction. The crude extract was filtered and the marc was discarded while the filtrate was concentrated over steel pans in an oven at 45 °C for 48 h. After concentration by evaporation, the sample was scrapped off the pans onto a clean Aluminum foil, weighed, put in a ziplock, and kept at 4–8 °C. It was then used for the nanoparticles extraction using molecular grade silver nitrate purchased from Sigma Aldrich USA and following the standard methods with minor modifications [32]. By adding 1 ml of green tea extract (1 g/ml) to two 100 ml round bottom flasks each containing 49 ml of an aqueous solution containing 85 mg of silver nitrate, AgNPs were created (0.5 mM). The magnetic stir bar and cooling condenser were attached to the round-bottom flask. The reaction mixture was allowed to stand for 2 h at 85 °C, after which it was allowed to cool and centrifuged for 30 min at 9000 rpm. The resultant sediment was thoroughly cleaned many times in distilled deionized water before being baked in an oven for 12 h at 80 °C. This was measured out (50 mg), collected into 1.5 ml Eppendorf tubes, and then stored for later use at 4 °C using aluminum foil. The biosynthesized AgNPs were characterized by ultraviolet (UV)-visible spectroscopy; Fourier transforms infrared spectroscopy (FTIR-1000) Perkin Elmer, X-ray diffraction (XRD) and Field Emission Scanning electron microscopy (FESEM)-Carl Zeiss Sigma model (see Ref. [33] on expanded protocol).

### 2.2 Study design

This was an invivo study in which 60 adult male *BALB/c* mice (weighing 25.5 ± 3.3 g) were randomly assigned to six independent groups (n = 10). The mice were given standard rodent pellets procured from a local supplier and water (ad libitum). They were grown and bred in the research laboratory so they had enough time to climatize to the environment for 14 days [34]. All the laboratory animals were exposed to sufficient day and night over a 12 h interval respectively.

### 2.3 Induction of inflammation

This was done using the basic principle of increasing the linear-circumference of the rodent hind paw by subplantar injection of fresh egg albumin (a phlogistic agent) following previous studies [[35], [36], [37]]. In this study, each experimental rodent was injected with 0.5 ml/kg of fresh egg albumin on the subplantar surface of the right hind paw for induction of acute inflammation and then the animals were monitored for 36 h.

### 2.4 Experimental animals

Green tea AgNPs were reconstituted in deionized water to form colloidal suspensions of 3 varying concentrations i.e. 1%, 10%, and 100% w/v inline with standard toxicological guidelines to widen the spectrum [38] with modifications from Ref. [39] who used a constant volume of 10 ml/kg (Table 1). Each concentration was administrated to the corresponding group of the experimental animals at the dose of 0.3 mg/kg p.o as previously described in inflammatory experiments [40]. The medication was administered in the evening, at a time when the surrounding environment was quiet and relaxed (1800 h local East African Time).

**Table 1:** Details on experimental groups and treatment categories for the study.

Treatment description	Category
Group I had inflammation with high concentration (100% w/v) of extract-AgNPs	High concentration
Group II had inflammation with medium concentration (10% w/v) of extract-AgNPs	Medium concentration
Group III had inflammation with a low concentration (1% w/v) of extract-AgNPs	Low concentration
Group IV had inflammation with diclofenac (comparative control) at 75 mg/kg	Comparative control
Group V had inflammation and was treated with the vehicle i.e. de-ionized water	Positive control
Group VI consisted of healthy mice that continued on normal food and de-ionized water and no edema/inflammation	Negative control

### 2.5 Determination of changes in hind paw swelling of mice

Linear paw edema was examined for three days at 30 min, 1, 2, 4, 8, 16, and 32 h respectively. The measurements (in centimeters) for the right hind paw edema were compared to those of the contralateral, non-injected left hind paw circumference. Inflammation (edema) index was quantified calculated using C_0_/C_T_ × 100 and presented as a percentage, while percent inhibition of edema was determined with the formula: C_0_– C_0_/C_T_ × 100 (Where C_0_ is the initial paw circumference; and C_T_ is the average inflammation of the extract or diclofenac treated mice at a specific time; t) in line with standard methods [37].

### 2.6 Determination of pain perception in the hind paw of the mice

#### 2.6.1 Voluntary wheel running

Voluntary wheel running was measured using wireless low-profile running wheels (Med Associates, Fairfax, VT). To minimize the effects due to environmental novelty, mice were first acclimated to the machine by making them run for 2 h/day for 2 days. Distance traveled was recorded for 2 h without the presence of the experimenter in the room. Non-runners mice are those which ran less than 100 m during the baseline session. They were identified and excluded from the post-injury voluntary wheel running test and all experiments were conducted between 8 a.m. and 12 p.m. [41].

#### 2.6.2 Locomotor activity and anxiety-like behavior (open field)

This was used to quantify locomotion in an open field chamber supplied with a digital camera under laboratory light. After acclimation, mice were separately put into the centre of the chamber and allowed to freely explore it for 1 h once the experimenter had left the room after induction of edema. The total distance traveled and time spent moving were calculated for the 42 L × 42 W × 20H cm chamber.

The percentage of time spent in the middle zone of the open field arena throughout the 1 h testing period was used to evaluate the level of anxiety-like behaviour. A square with 40% of the arena’s area was designated as the central zone. Mice were only evaluated in the open field once because open field activity is influenced by the inquisitive behavior of a novel setting. As a result, evaluation of the open field activity at various post-treatment timepoints was done using distinct cohorts of mice [41].

#### 2.6.3 Determination of temperature perception in mice

The thermal nocifensive response was measured using an incremental hot/cold plate (IITC, Woodland Hills, CA) in accordance with the temperature at which a mouse demonstrated a positive stimulus-response behavior, which was set in the current investigation as licking either hind or fore paw. Each mouse was placed separately on a hot plate set to 30 °C (a non-toxic temperature) within a transparent acrylic cylinder. A ramp rate of 5 °C per minute was used to raise the temperature. Once the mouse licked its paw or the hot plate reached the machine-set safety cutoff temperature of 50 °C, the test was declared over by the observer. The reading was accurate to within 0.1°. Two tests were conducted on each mouse, and the average was determined [42].

#### 2.6.4 Statistical analysis

Data analysis was done using Origin version 2019b on characterization while musculoneural activity datasets were exported to Graph Pad Prism Version 5 for analysis. Information was displayed as graphs and a table. Multiple Tukey’s tests were conducted between groups to decide the sources of variation and significance was considered when P < 0.05.

## 3 Results

### 3.1 Characterization of the green tea AgNPs

**Fig. 1:**
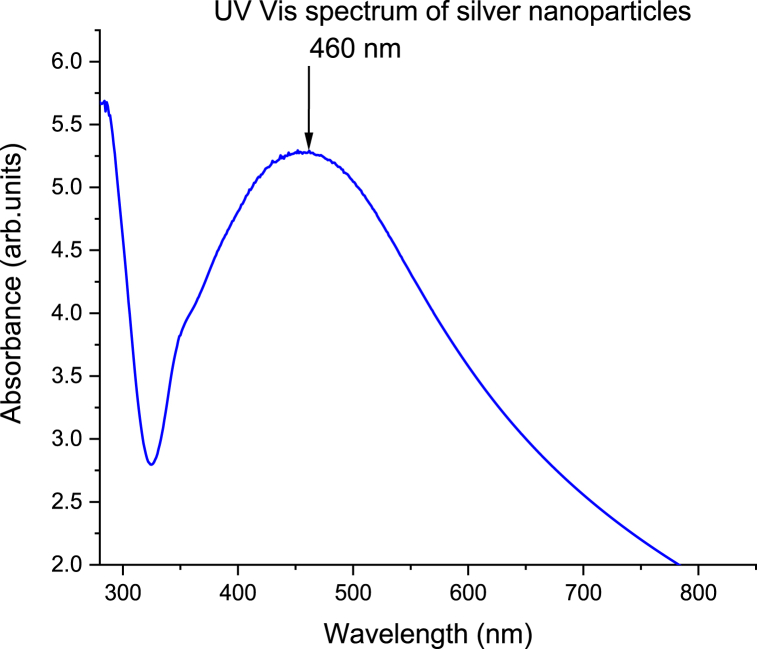
UV spectrum on the synthesized nanoparticles.

**Fig. 2:**
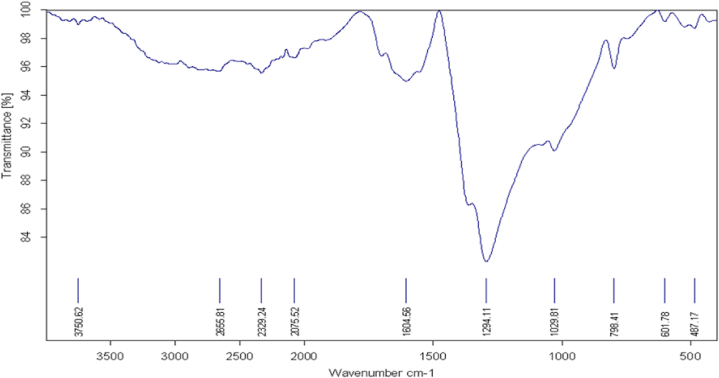
FTIR spectroscopy analysis detected organic functional groups in the wavenumber range of 2400–2000 cm^−1^, 1650-1600 cm^−1^, 1290 cm^−1^ and 1029 cm^−1^.

**Fig. 3:**
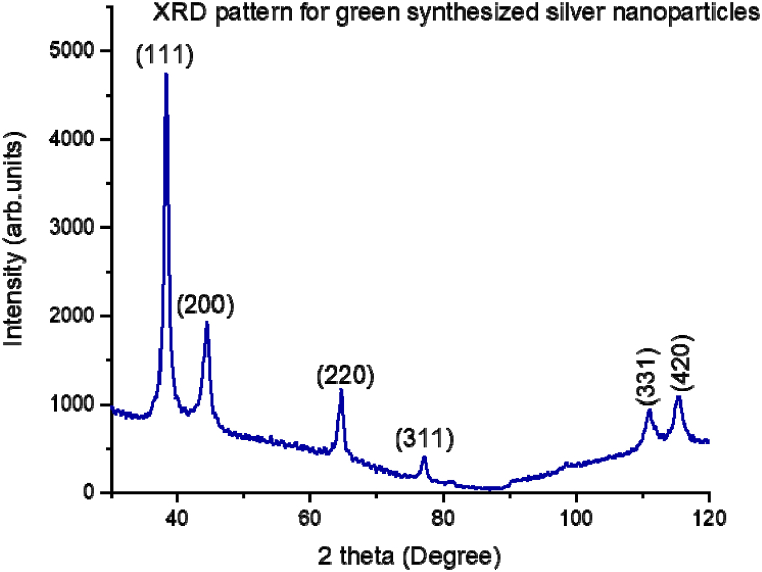
The XRD pattern had six 6 strong peaks at 2 theta angles of 38.1°, 44.2°, 64.4° 77.5°, 110.5° and ° 114.9° and the angles correspond to crystal planes (111), (200), (220), (311), (331) and (420) respectively.

**Fig. 4:**
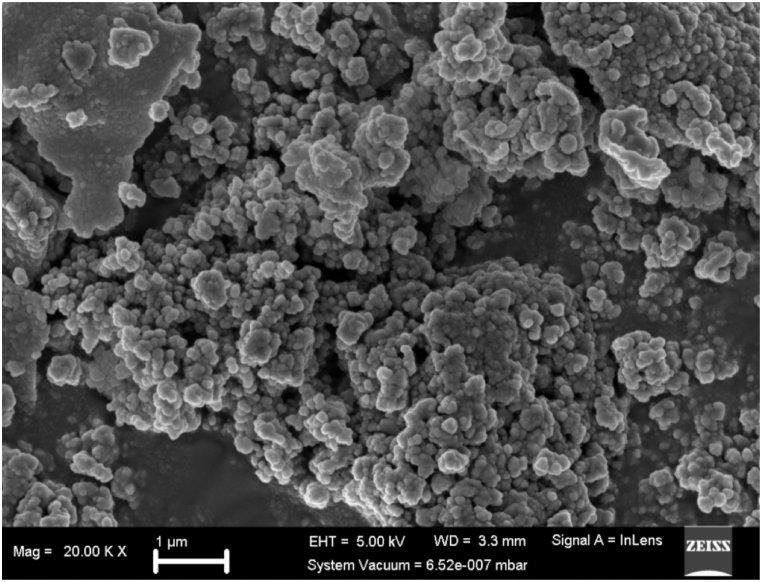
Morphological analysis by FESEM revealed that the biosynthesized AgNPs were mainly spherical and agglomerated in layers. ImageJ was used to estimate the nanoparticle size the AgNPs from FESEM images. The size distribution of the AgNPs ranged from approximately 2 nm–97 nm with average size of 8 nm.

The optical property of AgNPs was assessed by a UV Vis spectrophotometer. The absorbance band was observed at 460 nm (Fig. 1), while FTIR detected organic groups in 4 wave number ranges (Fig. 2). Furthermore, XRD patterns revealed six peaks (Fig. 3), while morphological analysis our AgNPs showed spherical and agglomerated layers (Fig. 4).

### 3.2 Changes in pain perception of white male mice following exposure to green tea nanoparticles

**Fig. 5:**
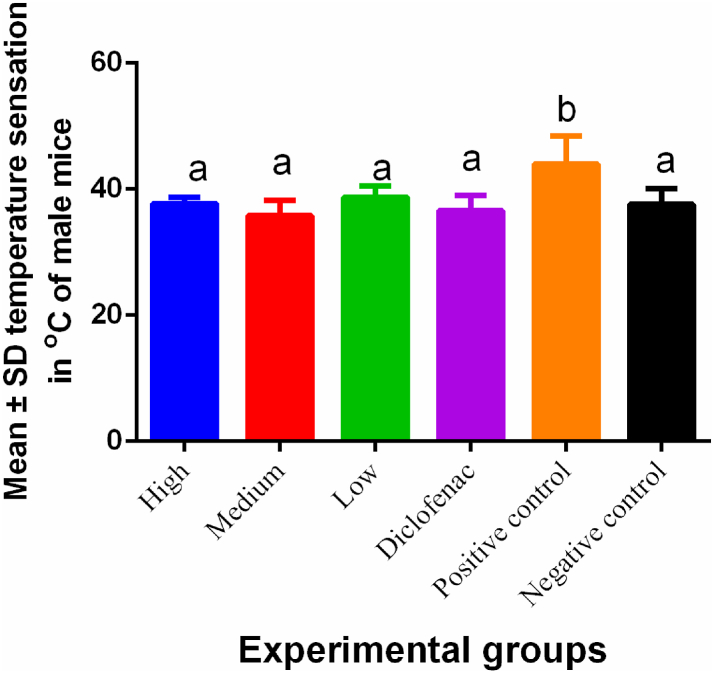
Mean temperature changes in mice following inflammation. Green tea nanoparticles demonstrated therapeutical effect by improving on neural sensation in affected mice while non-treated mice lost the ability to detect minute changes in temperature. Different superscripts (a,b) indicate significant differences (P < 0.05). (For interpretation of the references to colour in this figure legend, the reader is referred to the Web version of this article.)

The neural temperature threshold was significantly the same in all the mice treated with the green tea nanoparticles and diclofenac (Fig. 5). The positive control (no intervention group) demonstrated significant (P < 0.05) desensitization to the heat stimulus in the hind paw (Table 2).

**Table 2:** Multiple comparisons table showing adjusted P values.

Tukey’s multiple comparisons test	Temperature	Oedema	Anxiety	Distance
	Adjusted p values			
High vs. Medium	0.8589	0.9993	0.8911	<0.0001
High vs. Low	0.9899	0.6240	>0.9999	<0.0001
High vs. Diclofenac	0.9846	0.2230	0.9900	<0.0001
High vs. Positive control	0.0111	0.9066	0.6856	<0.0001
High vs. Negative control	>0.9999	0.1198	0.3933	0.0003
Medium vs. Low	0.5186	0.4135	0.8144	0.8540
Medium vs. Diclofenac	0.9964	0.1149	0.9971	0.4390
Medium vs. Positive control	0.0007	0.9827	0.9985	<0.0001
Medium vs. Negative control	0.8838	0.2311	0.9496	0.0498
Low vs. Diclofenac	0.8019	0.9775	0.9696	0.9804
Low vs. Positive control	0.0436	0.1253	0.5776	0.0032
Low vs. Negative control	0.9846	0.0023	0.3018	0.0021
Diclofenac vs. Positive control	0.0022	0.0235	0.9526	0.0227
Diclofenac vs. Negative control	0.9899	0.0003	0.7616	0.0002
Positive control vs. Negative control	0.0096	0.6101	0.9967	<0.0001

### 3.3 Changes in edema in white male mice following green tea nanoparticles administration

**Fig. 6:**
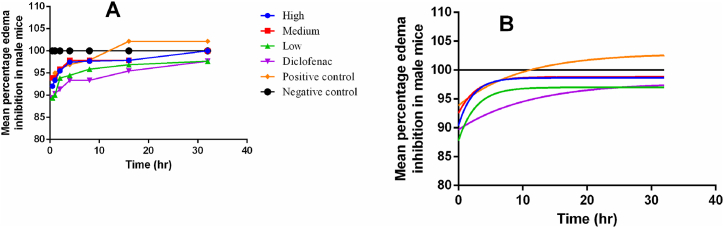
Edema inhibition following exposure to nanoparticles. A = changes following experimental exposure. B = smooth curves following nonlinear regression). Generally, all mice recovered well after 24hr although those exposed to the medium concentration of 10% nanoparticles performed better.

The study showed that low concentrations of silver-tea-nanoparticles inhibited edema as efficient as diclofenac. However, percentage inhibition was higher in medium and high silver-tea-nanoparticles concentrations. Moreover, no changes in edema were associated with the negative control (Fig. 6A and B). The nonlinear regression analysis showed that the half-life of one phase decay for high, medium, low, diclofenac, positive control, and negative control was in the order of (half-life ± 95% CI, *R*^*2*^) of 1.532 ± 0.7665–1063 h, 0.9358; 1.917 ± 0.8268 to + infinity h, 0.8955; 1.851 ± 0.9939–13.40 h, 0.9523; 7.664 ± 3.619 to + infinity h, 0.9508; 6.750 ± 3.497–96.84 h, 0.9616; and ∼2.773 ± 0.00 h, 0.9616 respectively (Table 1).

### 3.4 Changes in locomotion of white male mice following tea nanoparticles exposure

**Fig. 7:**
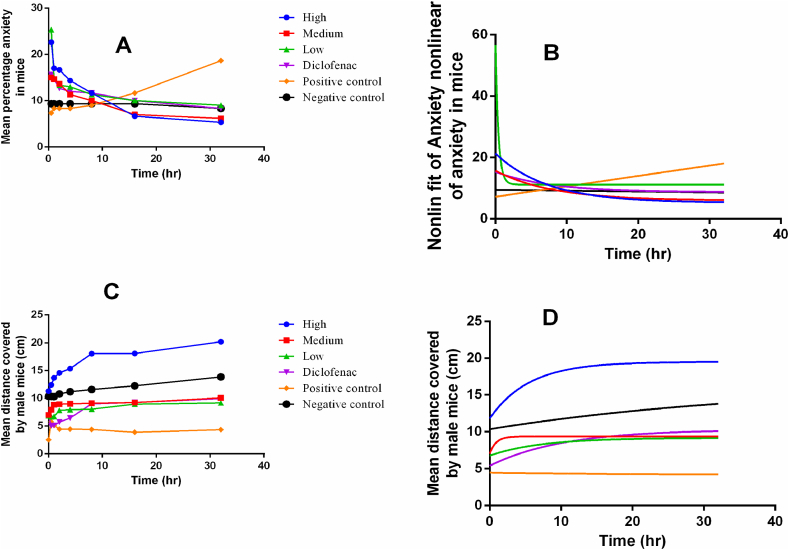
Changes in anxiety and covered distance by mice with nanoparticles administration.

High concentrations of green tea nanoparticles decreased anxiety rapidly, followed by the medium and low concentrations while the positive control was associated with elevated levels of anxiety as shown in Fig. 7A. High concentrations of green tea nanoparticles were associated with an increase in the distance covered which was significantly greater than that of the medium, low, and diclofenac groups. The movement in the negative control was moderately above average as shown in Fig. 7C. Inflammation increased anxiety in the mice while treatment with green tea nanoparticles was associated with a significant decrease in anxiety although this was dose-independent as shown in Fig. 7A and B. An increase in anxiety was associated with a reluctance to move thus decreasing locomotion behaviour (Fig. 7C and D).

## 4 Discussion

Chronic inflammatory diseases are common and these expose patients to life-threatening complications following long-term use of the anti-inflammatory therapies usually manifested in the form of renal failure and gastric ulcers [2,3]. The use of aqueous green tea extract is cheaper, more readily available, and has less side effects as compared to conventional anti-inflammatory therapies and it can also improve the individuals health [22], especially in resource limited countries.

In this study, we successfully applied green synthesized AgNPs using *Camellia sinensis* extract. *Camellia sinensis* contains catechins with potent antioxidant activity; thus, reduce inorganic metal ions to nanoscale form [33] and this was in agreement with previous studies in which morin which is common in flavonoids exhibited the same properties [21]. This was confirmed by UV Vis spectroscopy that registered absorbance band at 460 nm which is a unique optical property of AgNPs [43]. The phytochemicals that bio-reduce metal ion to nanoscale stabilize and functionalize the resultant nanoparticles by capping. This was confirmed by FTIR analysis that detected the presence of organic functional groups in wavenumber range of 2400–2000 cm^−1^ representing O=C=O of oxycarbons, 1650-1600 cm^−1^ signifying the presence of C=C of a conjugate alkene, 1290 cm-1 for amino groups, 1029 cm^−1^ C=O of a stretching bond of a secondary alcohol confirming capping of AgNPs by the phytochemicals used in the bio-reduction of non-nanoscale silver to nanoform.

In our previous study [33] and current study, we confirm that The XRD analysis showed six strong peaks at 2 theta angles of 38.1°, 44.2°, 64.4° 77.5°, 110.5°, and ° 114.9°, respectively, corresponding to crystal planes (111), (200), (220), (311), (331) and (420). The recorded XRD pattern crystal lattice planes corroborate with the standard values deposited in the International Centre for Diffraction Data (ICDD) database, file number 00-004-0783. SEM analysis revealed that indeed the phytochemicals in *Camellia sinensis* extract bio-reduced silver nitrate to the nanoscale level as the average size distribution of the nanoparticles ranged from 2 nm to 97 nm with average size of 8 nm. SEM image show that the nanoparticles were spherical covered by a slimy layer of the phytochemicals further confirming capping and stabilization of the nanoparticles [33].

In the current study, AgNPs significantly lowered the temperature hypersensitivity in *BALB/c* male mice, thus demonstrating their anti-inflammatory activity. During inflammation, elevated temperature response is characteristic of cellular injury demonstrating the importance of our findings. In addition, low concentrations of green tea AgNPs inhibited edema as efficient as diclofenac. However, percentage inhibition was highest in high and medium green tea AgNPs concentrations while no edema associated changes were observed in the negative control. This was important since swelling is a cardinal sign of inflammation, which is usually associated with pain. Our findings are in agreement with Chandra et al. [44] who also demonstrated the importance of AgNPs. During swelling, pro-inflammatory cytokines contribute to the increase in the size of the inflamed site following ischemic injury or trauma through endothelial cells destruction. Green tea extracts attenuate endothelial cells swelling through the reduction of Ca^2+^ [45]. Nonlinear regression showed that the half-life of one phase decay was in the order of high > negative control > medium > low > diclofenac > positive control demonstrating that green tea AgNPs at high concentrations would have a higher duration (T1/2) in the body. This was important since the quality of green tea AgNPs has been associated with elevation of cultivation [44]. Here, we provide evidence on the importance of green tea AgNPs cultivated in a tropical country of East Africa.

During inflammation, swelling and pain often occur together and these are associated with a decrease in exercise tolerance and function in the affected limb [14]. Locomotory behavior involving movement of mice from one place to another (physiological behavior that was disrupted by the presence of pain sensation) was disrupted due to the intensity and duration of the stimuli [15]. In our study, we demonstrate the potential therapeutical advantage associated with green tea AgNPs for adoption in complementary and alternative medicine once further preclinical and clinical studies are conducted in Uganda. In animals, pain subsequently raises the level of anxiety causing nervousness, restlessness, fear of moving from one place to another, and pervading a sense of unrealistic worry about everyday life situations [15]. High concentrations of green tea nanoparticles rapidly decreased the anxiety levels, and this was associated with the increased ability by the mice to move and explore their environment. Our study was in agreement with a previous study which reported that a decrease in anxiety was associated with the increase in the distance covered in mice [16]. The performance of medium and low concentrations of the green tea AgNPs was associated with moderate physiological changes in *BALB/c* male mice, demonstrating that pharmacodynamic effects associated with green tea AgNPs are dose and concentration dependent. In the positive control, an increase in anxiety was associated with a reluctance to move thus decreasing the locomotion behavior. The results of this study offer further evidence on the use of nanoparticles as drug delivery options in pharmacognosy related studies. In addition, severe side effects associated with the conventional anti-inflammatory therapies would be reduced through the adoption of complementary medicines in nanomedicine.

## 5 Conclusion

The study showed that green tea AgNPs have strong anti-inflammatory effects at high concentrations due to their strong phytochemical and physiochemical properties. In addition, the various concentrations of green tea AgNPs modified basic sensory and motor behaviors in *BALB/c* male mice demonstrating their importance in complementary and integrative medical practice. Further studies on the role played by green tea AgNPs in mice in the absence of inflammation could offer further insights on cellular pathways of concern. Molecular pathways especially the recruitment of inflammatory and anti-inflammatory mediators, gene expression and cellular signaling remain to be investigated.

## Author contribution statement

Herbert Izo Ninsiima, Caroline Asekenye, David Onanyang, Moses Ariong, Kevin Matama, Halima Nalugo, Adam Moyosore Afodun, Regan Mujinya: Performed the experiments; Contributed reagents, materials, analysis tools; Wrote the paper.

Ejike Daniel Eze, Kenneth Ssekatawa, Edson Ireeta Munanura, Gerald Zirintunda, Ngala Elvis Mbiydzenyuy, Ibe Michael Usman, Julius Tibyangye: Analyzed and interpreted data; Contributed reagents, materials, analysis tools; Wrote the paper.

Fred Ssempijja, Oscar Hilary Asiimwe: Performed the experiments; Analyzed and interpreted the data; Contributed reagents, materials, analysis data; Wrote the paper.

Keneth Iceland Kasozi: Conceived and designed the study; Performed the experiments; Analyzed and interpreted the data; Contributed reagents, materials, analysis tools; Wrote the paper.

## Funding statement

Kenneth Ssekatawa was supported by Africa Centre of Excellence in Materials, Product Development & Nanotechnology [P151847IDA].

## Data availability statement

Data associated with this study has been deposited at FigShare: https://figshare.com/s/f2312c0c5e5c0378be8d.

## Declaration of interest’s statement

The authors declare no competing interests

## Declarations

Ethical approval and consent to publish.

The study was approved by a scientific review board of Kampala International University Western Campus, consisting of biomedical scientists who approved the study for implementation. Consent to participate was not applicable to this study.
